# Carbon ion irradiation combined with PD-1 inhibitor trigger abscopal effect in Lewis lung cancer via a threshold dose

**DOI:** 10.7150/jca.91559

**Published:** 2024-02-25

**Authors:** Ruifeng Liu, Yichao Geng, Hongtao Luo, Qiuning Zhang, Zhen Yang, Shan Li, Shilong Sun, Zhiqiang Liu, Meng Dong, Tianqi Du, Tuanjie Che, Xiaohu Wang

**Affiliations:** 1 Institute of Modern Physics, Chinese Academy of Sciences, Lanzhou, China.; 2 The First School of Clinical Medicine, Lanzhou University, Lanzhou, China.; 3 The affiliated cancer hospital of Guizhou Medical University, Guiyang, China.; 4 Heavy Ion Medicine Center, Hospital of economic and Technological Development Zone, Wuhan Renmin Hospital of University, Wuhan, China.; 5 Gansu University of Traditional Chinese Medicine, Lanzhou, China.; 6 Key Laboratory of Functional Genomics and Molecular Diagnostics of Gansu Province, Lanzhou, China.

**Keywords:** Carbon ion, Irradiation, PD-1 inhibitor, In vitro experiment, Abscopal effect

## Abstract

**Background and goal:** Carbon ion beam is radio-biologically more efficient than photons and is beneficial for treating radio-resistant tumors. Several animal experiments with tumor-bearing suggest that carbon ion beam irradiation in combination with immunotherapy yields better results, especially in controlling distant metastases. This implies that carbon ion induces a different anti-tumor immune response than photon beam. More complex molecular mechanisms need to be uncovered. This in vivo and in vitro experiment was carried out in order to examine the radio-immune effects and the mechanism of action of carbon ion beam versus X-ray in combination with PD-1 inhibitors.

**Methods and Materials:** Lewis lung adenocarcinoma cells and C57BL/6 mice were used to create a tumor-bearing mouse model, with the non-irradiated tumor growing on the right hind leg and the irradiated tumor on the left rear. 10Gy carbon ion beam or X-ray radiation, either alone or in combination with PD-1 inhibitor, were used to treat the left back tumor. The expression of molecules linked to immunogenicity and the infiltration of CD8+ T lymphocytes into tumor tissues were both identified using immunohistochemistry. IFN-β in mouse serum was measured using an ELISA, while CD8+ T cells in mouse peripheral blood were measured using flow cytometry. Lewis cells were exposed to different dose of X-ray and carbon ion. TREX1, PD-L1, and IFN-β alterations in mRNA and protein levels were identified using Western blot or RT-PCR, respectively. TREX1 knockdown was created by siRNA transfection and exposed to various radiations. Using the CCK8 test, EdU assay, and flow cytometry, changes in cell viability, proliferation, and apoptosis rate were discovered.

**Results:** Bilateral tumors were significantly inhibited by the use of carbon ion or X-ray in combination with PD-1, particularly to non-irradiated tumor(p<0.05). The percentage of infiltrating CD8+ T cells and the level of IFN-β expression were both raised by 10Gy carbon ion irradiation in the irradiated side tumor, although PD-L1 and TREX1 expression levels were also elevated. Lewis cell in vitro experiment further demonstrated that both X-ray and carbon ion irradiation can up-regulate the expression levels of PD-L1 and TREX1 with dose-dependent in tumors, particularly the trend of up-regulation TREX1 is more apparent at a higher dose in carbon ion, i.e. 8 or 10Gy, while the level of IFN-β is decreased. IFN-β levels were considerably raised under hypofractionated doses of carbon ion radiation by gene silencing TREX1.

**Conclusions:** By enhancing tumor immunogenicity and increasing CD8+T infiltration in TME through a threshold dosage, X-ray or carbon ion radiation and PD-1 inhibitors improve anti-tumor activity and cause abscopal effect in Lewis lung adenocarcinoma-bearing mice. TREX1 is a possible therapeutic target and prognostic marker.

## Introduction

In vitro and animal studies have clearly shown benefits of radiotherapy (RT) plus immune checkpoint inhibitor (ICI) in comparison to ICI alone. The most significant and widely used ICI in non-small cell lung cancer (NSCLC) are those targeting the proteins programmed cell death protein-1 and programmed cell death ligand-1 (PD-1/PD-L1) 1. In recent years, clinical trials are increasingly being performed to investigate the role of combination therapy using PD-1/PD-L1 inhibitors and RT for NSCLC^2^, the pooled results indicated combination therapy using PD-1/PD-L1 inhibitors and RT may improve overall survival (OS), progression-free survival (PFS), and tumor objective response rates (ORR) without an increase in serious adverse events in patients with advanced NSCLC^3^. ICD, or radiation-induced immunogenic cell death, is a critical step in the beginning of the immune response against the tumor. Irradiated cancer cells have increased immunogenicity, which phagocytes pick up and convert from non-immunogenicity to immunogenicity to mediate the anti-tumor immune response, or ICD. The primary mechanism of ICD was that radiation increased the production of antigens linked with tumor cells that may be detected by T lymphocytes 4.

Clinical studies have also demonstrated that radiotherapy not only leads to the expansion of CD8+T cells in vivo, but also induces the expression of new tumor-associated antigens, which are recognized by CD8+T cells^5^.Priming and activation of CD8+T cells causes a systemic immune response against tumor tissue, both irradiated or unirradiated 6,7, this phenomenon of radiotherapy leading to tumor response and control in non-irradiated areas has been described as a abscopal effect. Although this phenomenon was first described by Mole in 1953, it only recently garnered revived clinical attention 8. One explanation for this is that achieving an abscopal effect may be simpler with immunotherapies plus radiation, as hypothesized based on recent advancements in immunotherapy.

Although the occurrence of the abscopal effect is rather rare, the combination of RT and immunotherapy may enhance its frequency and hence improve prognosis. Data from large randomized trials are currently mainly absent, and the majority of data come from case reports with relatively small sample sizes. The addition of radiation to pembrolizumab has been shown to dramatically increase response rates in unirradiated tumors, leading to a significant improvement in PFS and OS, as indicated by a pooled study of 148 patients. The combination of pembrolizumab with radiotherapy could be considered a treatment option for patients with metastatic NSCLC 9. The findings of this study further increase the confidence in employing radiation in conjunction with ICI to generate the abscopal effect in future research. Uncertainty exists over the ideal mixtures to produce an abscopal effect. Other investigations need to take into account four key issues: RT settings, therapeutic sequencing, the abscopal effect definition, and patient selection. Among these, it's crucial to look into fresh molecular targets or use cutting-edge radiation to boost the effectiveness of this combined treatment approach.

When combined with PD-1 inhibitors, carbon ion therapy may be more effective at treating NSCLC because it produces complex cluster damage in tumor cells' DNA that is primarily composed of double-strand breaks and has a higher relative biological effectiveness (RBE) than X-ray. Several studies of animal experiment in tumor-bearing suggest that carbon ion beam irradiation in combination with immunotherapy yields better results, especially in controlling distant metastases, than either alone or photon combined with ICI^10^,^11^,^12^. Furthermore, both cell line and animal experiments have confirmed that heavy ion can increase the expression of ATP, HMGB1 and CRT DAMPs more than photon radiation, thus inducing stronger ICD in tumor cells^13^,^14^.This suggests that heavy ion beam induces a different anti-tumor immune response than photon beam, more complex molecular mechanisms need to be further explored.

Several studies have shown that TREX1, IFN-β, and other biomarkers are the important molecules in radiation-induced ICD and immune response. In the studies of murine colon cancer and human breast cancer cell lines, the upregulation of TREX1 inhibits the activation of c-GAS, and inhibition of TREX1 expression in cancer cells can enhance anti-tumor immune response. In the process of cell carcinogenesis, type I IFN produced by activation of the cGAS-STING pathway provides a key signal for anti-tumor immunity. TREX1 can maintain the innate immunity of the host and the tolerance of cells to their own DNA under homeostatic and genotoxic stress by removing the accumulated accessory substance of DNA damage in the cytoplasm. Cancer cells utilize this mechanism of TREX1 action to inhibit the activation of cGAS-STING caused by DNA damage and thus realize immune escape. But how TREX1 and cGAS-STING pathways function in NSCLC has not been confirmed by relevant studies.

To provide a theoretical foundation for the treatment of NSCLC patients with X-ray and carbon ion combined with ICI, we conducted an in-vitro experiment to investigate the radio-immune effects and its mechanism of different LET rays combined PD-1 inhibitors (murine PD-1) to irradiated and abscopal tumors on lung adenocarcinoma.

## Materials and Methods

### Cell line

Human Lewis NSCLC cell were purchased from ATCC (American type culture collection). Cell line was cultured in Dulbecco's modified eagle's medium (DMEM) supplemented with 10% FBS. All cell line were verified as being free of microbial contamination.

### Preparation of cell suspension

Lewis cells' growth morphology was studied using an electron microscope. On the spotless workstation, the culture media was carefully poured, and 3cc of room-temperature PBS was gently cleaned twice before being discarded. After a gentle shake to distribute the pancreatic enzymes, 1 ml of EDTA enzyme was added, and the cells were then incubated at 37^ o^C for 2 min. Under an electron microscope, the morphology of the cells was studied, and trypsin was decanted when the cells shrank into thin slices and became brilliant and rounded. After stopping digestion, 3ml of culture medium containing FBS was added, and a homogeneous cell suspension was created by gently and frequently blowing air through a sterile straw.

### Experimental animals

C57BL/6 mice, male, weighing 16-17g, aged 21-28 days, were purchased from the SPF Animal Laboratory of Gansu University of Traditional Chinese Medicine. Feeding conditions and environment as follow. 6 mice per cage, temperature was 24±2°C, humidity was 50%-70%, the light was given by 12h-12h intermittent day and night illumination, feed was added once every two days, water was changed once a day, and bedding material was changed once every three days. The mice ate and drank freely throughout.

### Establishment of transplanted tumor model

**Figure 1:**
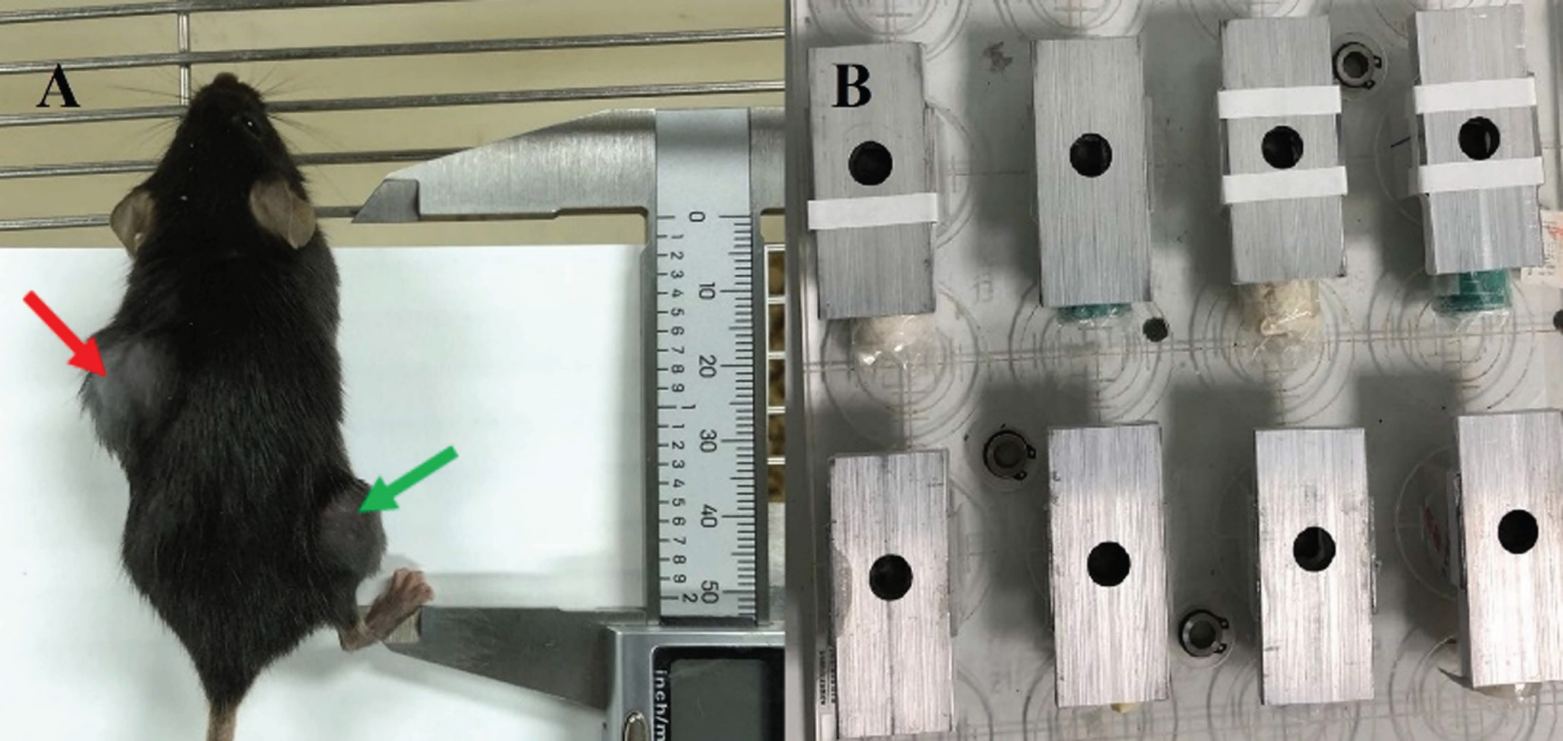
A was C57BL/6 mice with bilateral tumor bearing models, the left back was an irradiated area (indicated by the red arrow) and the right hind limb was a non-irradiated area (indicated by the green arrow). B were homemade irradiation-shielded lead boxes for mice.

To create the irradiation tumor model, the left back of mice was chosen, and the injection site was sterilized with 75% alcohol on the ultra-clean surface. The homogenous cell suspension was absorbed using a sterile 1 ml syringe and injected subcutaneously on the left back after being thoroughly blasted with a pipette gun prior to injection. With a concentration of 1×10^6^ cells per mouse, the inoculation volume was 0.1 ml, and the inoculation density varied depending on the cell. On the right leg, the non-irradiated tumor model was implanted subcutaneously. The inoculum contained 1×10^5^ cells per mouse and had a volume of 0.1 ml. See Fig. 1.

### Mice allocation

7 days after inoculation, when the average volume of irradiated tumor reached about 140mm^3^ and the non-irradiated tumor reached about 70mm^3^, mice were randomly allocated into control group, X-ray irradiation group (X group), X-ray irradiation combined with αPD-1 inhibitor group (X+αPD-1 group), Carbon ion irradiation group (C group), Carbon ion irradiation combined with αPD-1 inhibitor group (C+αPD-1 group) and αPD-1 inhibition group (αPD-1 group), 6 mice in each group.

### Irradiation and drug administration

Carbon ion (^12^C^6+^) beam irradiation was performed at the treatment terminal of the Heavy Ion Research Facility in Lanzhou (energy, 80 MeV/u; peak LET, 50 KeV/μm; SOBP, 5mm; irradiation field, 5x5cm). X-ray were generated by an X-Rad 225 generator (Precision) (energy, 225 KV/13.3 mA).

**Figure 2:**
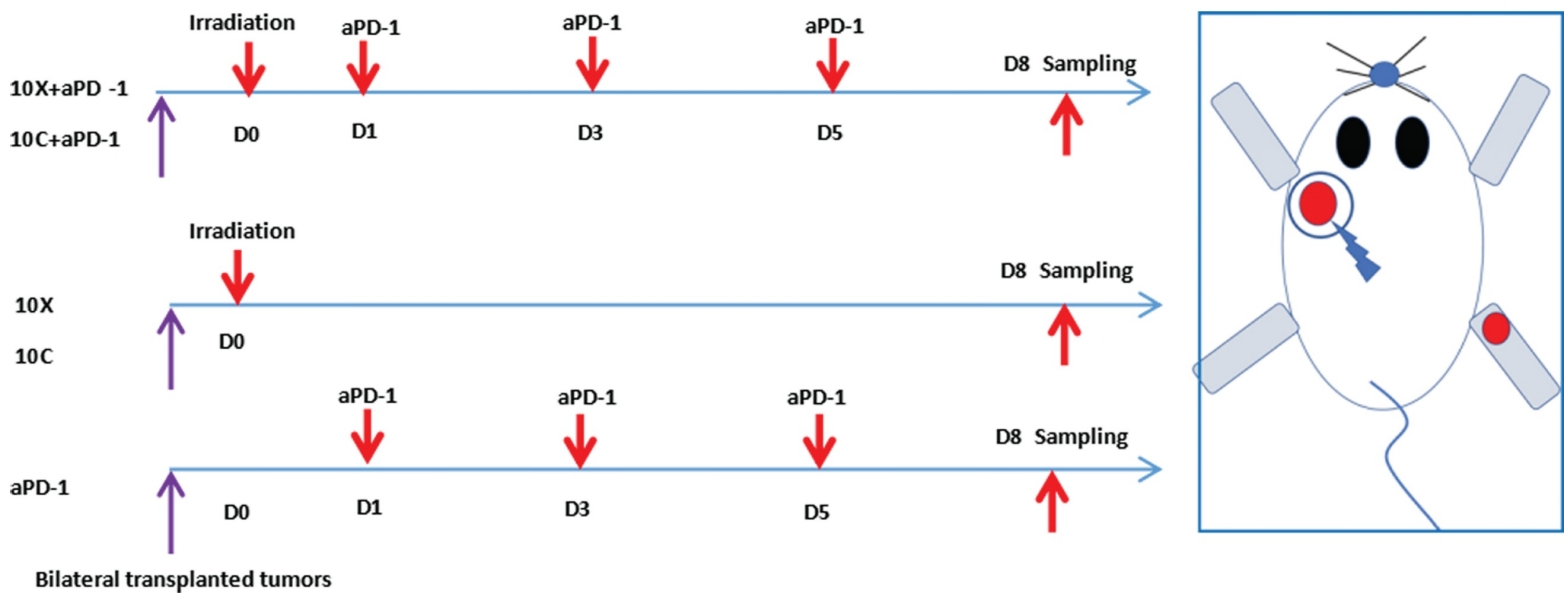
Schematic diagram of tumor irradiation and aPD-1 intervention in tumor-bearing mice.

After anesthesia, tumor-bearing mice were exposed to radiation. Each mouse was placed into a 50mL centrifugal tube for immobilization, then put them into homemade lead boxes. The tumors in the irradiated areas were exposed to the carbon ion beam through the window reserved on the lead box, and the non-irradiated areas were shielded by the lead box (see Figure 1). The mice in the matching radiation group received 10Gy of X-ray or carbon ion beam radiation to the left back tumor. (View Fig. 1-2). Lewis lung cancer tumor-bearing mice were administered intraperitoneally with murine PD-1 inhibitor (Bioxcell) at a dosage of 25 mg/kg/time, once every 2 days for a total of 3 times, diluted with 0.9% sterile saline. For combination treatment, Day0 (also known as D0) was chosen as the irradiation day, and PD-1 inhibitor was administered three times on Day 1, 3, and 5, as shown in Fig. 2.

The morphology of the cells was assessed prior to cell line irradiation, and the required number of cells were evenly plated in a 60-well culture dish and incubated for 24 hours in a cell incubator to cause them to adhere to the wall for growth. To identify the alterations in the target molecules, the two types of rays received the same radiation dosage and were exposed to 0Gy, 2Gy, 4Gy, 6Gy, 8Gy, and 10Gy, respectively. The TREX1 gene was then knocked out using siRNA to produce siTREX1-Lewis cells, which were irradiated with 2Gy and 10Gy, respectively, for various following detection methods. Three times each operation was carried out at room temperature.

### Tumor observation and evaluation

C57BL/6 mice with tumors were typically fed in a sterile setting after injection. Every two days during the procedure, each mouse's tumor diameter and weight were assessed using a vernier caliper and a weighing scale. The collected values were used to compute the tumor volume and weight. Mice with tumor volumes less than 1500 mm^3^ were cervical dislocated to death at day eight following radiation treatment in accordance with the features of the Lewis tumor, animal welfare standards, and the goals of the investigation. Complete subcutaneous dissection of bilateral tumors allowed for accurate measurement of their weight and volume. Save the remaining tumor tissue using 4% paraformaldehyde fixed liquid preservation, with the exception of few fresh tumor tissues needed in particular experiments.

### Immunohistochemistry (IHC) assay

Three-millimeter slices were cut from the paraffin blocks and put on positively charged slides for IFN-, TREX1, PD-L1, and CD8+T immunohistochemistry staining. The 3,3'-diaminobenzidine tetrahydrochloride (DAB) as the chromogen was employed in conjunction with the Lab VisionTM UltraVisionTM Quanto Detection System (#TL-060-QAL, Thermo Fisher Scientific, Waltham, MA, USA) and the primary antibody at a dilution of 1:200. Blue is considered to be negative, Light yellow is considered to be weakly positive, and Brownish yellow is considered to be considerably positive depending on the degree of color development of the positive cell labeling under an electron microscope. IHC measurement indications included the average gray value (staining intensity) and percentage of positive area (staining area) of positive cells 15. Four scores were ultimately assigned: strong positive was 3+, positive was 2+, weak positive was 1+, and negative was 0. Qualified pathologists using an electron microscope must see and evaluate the final tissues staining findings.

### ELISA

The quantification of circulating IFN-β was performed by means of enzyme-linked immunosorbent assay (ELISA), following the manufacturer's instructions. Absorbance (at 450 nm) was analyzed by means of an i3 Paradigm multimode platereader (Molecular Devices, SanJose, CA, USA). The IFN-β ELISA kits were purchased from solarbio (Beijing, China).

### Flow cytometry assay

**Figure 3:**
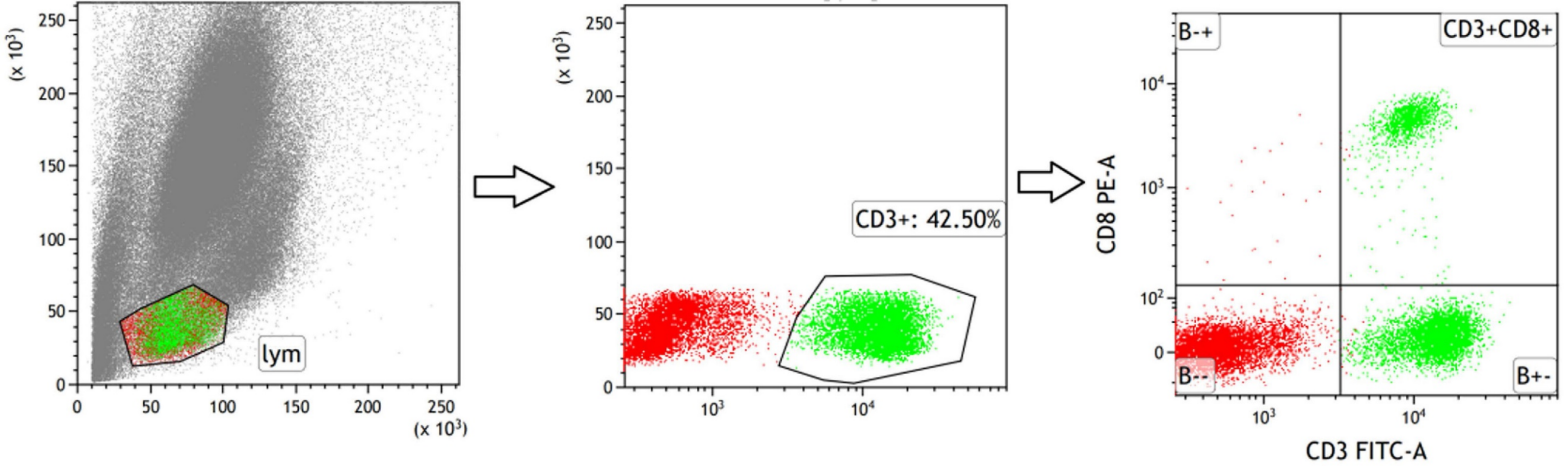
Strategies for sorting CD3+T and CD3+CD8+T subsets in peripheral blood of mice.

Blood was drawn from the eyeballs. The skin around the eye was carefully pressed and removed using ophthalmic tweezers while under the anaesthetic of 2% isoflurane. Anticoagulant tubes were used to collect the blood from the orbit, which was then immediately divided into two parts. One part underwent flow cytometric analysis of CD3+CD8+T cell sorting after being centrifuged at 4°C for 10 minutes. The supernatant from the second part was immediately stored at -80°C for later use. CD3e Monoclonal Antibody FITC and CD8a Monoclonal Antibody PE were purchased from Thermo Fisher (Massachusetts, USA). Flow cytometry was conducted using BD flow cytometer (the sorting strategies are shown in Fig. 3).

### Cell transfection experiment

Santa Cruz Biotechnology's transfection reagents were used in cell transfection experiment as directed. The particular techniques are as follows: The cultured cells were digested with trypsin to make a uniform cell suspension. First, 100µl of siRNA(sc-36868) transfection medium was added to 6µl (60 pmols) of siRNA(sc-63158), mixed, and designated as liquid A. The transfection medium siRNA (sc-36868) was then combined with 100µl of siRNA (SC-36868) and 6µl of siRNA (sc-29528) reagent to create liquid B. For the solution, liquids A and B were thoroughly combined and left at room temperature for 30 minutes. After thoroughly combining the liquid A and liquid B mixing tubes with the 0.8ml siRNA (sc-36868) transfection solution, the cell culture holes were filled. The cells were then cultivated in a CO_2_ incubator for 6 hours. After the cells had been examined under an electron microscope, 1ml of cell media with twice as much FBS and antibiotics was added right away. The cells were then cultivated for another 24 hours at a steady temperature in an incubator. The medium was then removed, and one standard medium was added for a further 24 hours. It may now be used to identify and analyze results from other intervention testing. Lewis was transfected into siNC-Lewis as the negative control group for later detection and analysis. The matching Control siRNA (sc-37007) was utilized to replace sc-63158 and repeat the same stages.

### Real-time quantitative RT-PCR and Western blotting

RNA was extracted using Trizol, and 3μg of RNA was used for reverse transcription in accordance with the instructions provided in the GeneCopoeia reverse-transcription kit handbook. SYBR Green was employed for real-time quantitative detection after the cDNA had been diluted three times. There were 40 cycles completed with a 20 μL final volume. Using a heating rate of 0.5°C/6 sec between 72°C and 95°C, predenaturation was carried out at 95°C for 10 min, denaturation at 95°C for 10 sec, annealing at 60°C for 20 sec, and primer template extension at 72°C for 15 sec. The Bio-Rad CFX96 PCR system software (Bio-Rad Laboratories, Hercules, CA, USA) was used to gather all the data. In order to examine target gene expression using the 2-CT approach, we calibrated the collected Ct values to glyceraldehyde 3-phosphate dehydrogenase (GAPDH) as an internal reference. The target gene primer sequence in the amplification system is shown in Table 1.

In RIPA buffer containing protease and phosphatase inhibitors, cells were lysed. The BCA assay kit (Termo Scientific, USA) was used to determine the protein concentration. Lysates were separated on a 10% or 15% SDS polyacrylamide gel after being denatured at 100 °C for 10 min. Proteins were loaded onto PVDF membranes from Millipore in the United States and blocked with BSA from Solarbio in China for 1.5 hours. TREX1, CD274, and IFNB1-specific antibodies were utilized. Using Image J software, all western blot signals were quantified.

### CCK‑8 assay

Cell viability was evaluated using a Cell Counting Kit-8 (CCK-8, APExBIO, USA). Cells were seeded at 3×10^3^ cells/ well in 96-well plates. Cells were irradiated 24 h after transfection. After 24h, 48h, 72h of treatment, 10 μL of CCK-8 reagent was added to every well for two hours. The optical density (OD) was measured at 450 nm.

### 5‑Ethynyl‑20‑deoxyuridine (EdU) incorporation assay

Te EdU incorporation assay was performed using a Cell Light EdU DNA Cell Proliferation Kit (RiboBio, China). Images were acquired with a fuorescence microscope (Olympus, Japan).

### Cell apoptosis assay

According to the requirements for concentration, cultured cells were injected into 6- and 12-well plates. The fluid was changed after the cells had been cultivated to be completely attached to the wall. The cell plates matching to each treatment were digested and turned into suspension after each dose was irradiated for 24 hours. Finally, flow cytometrywas used to measure the cell apoptosis rate.

### Statistical analysis

Unless otherwise stated, we will repeat the experiment three times. Spss19.0 software was used to analyze the data. Wilcoxon test was used to compare the mean values between groups, and Fisher's exact test was used to compare the survival fraction of irradiated cells. The data were analyzed and the histograms generated using GraphPad Prism 7 software. Statistical differences were determined by two-way analysis of variance (ANOVA), followed by Bonferroni test to compare treatment with control group (*p< 0.05, ** *p*<0.01, and ***p<0.001).

## Results

### Weight change of tumor-bearing mice

**Figure 4:**
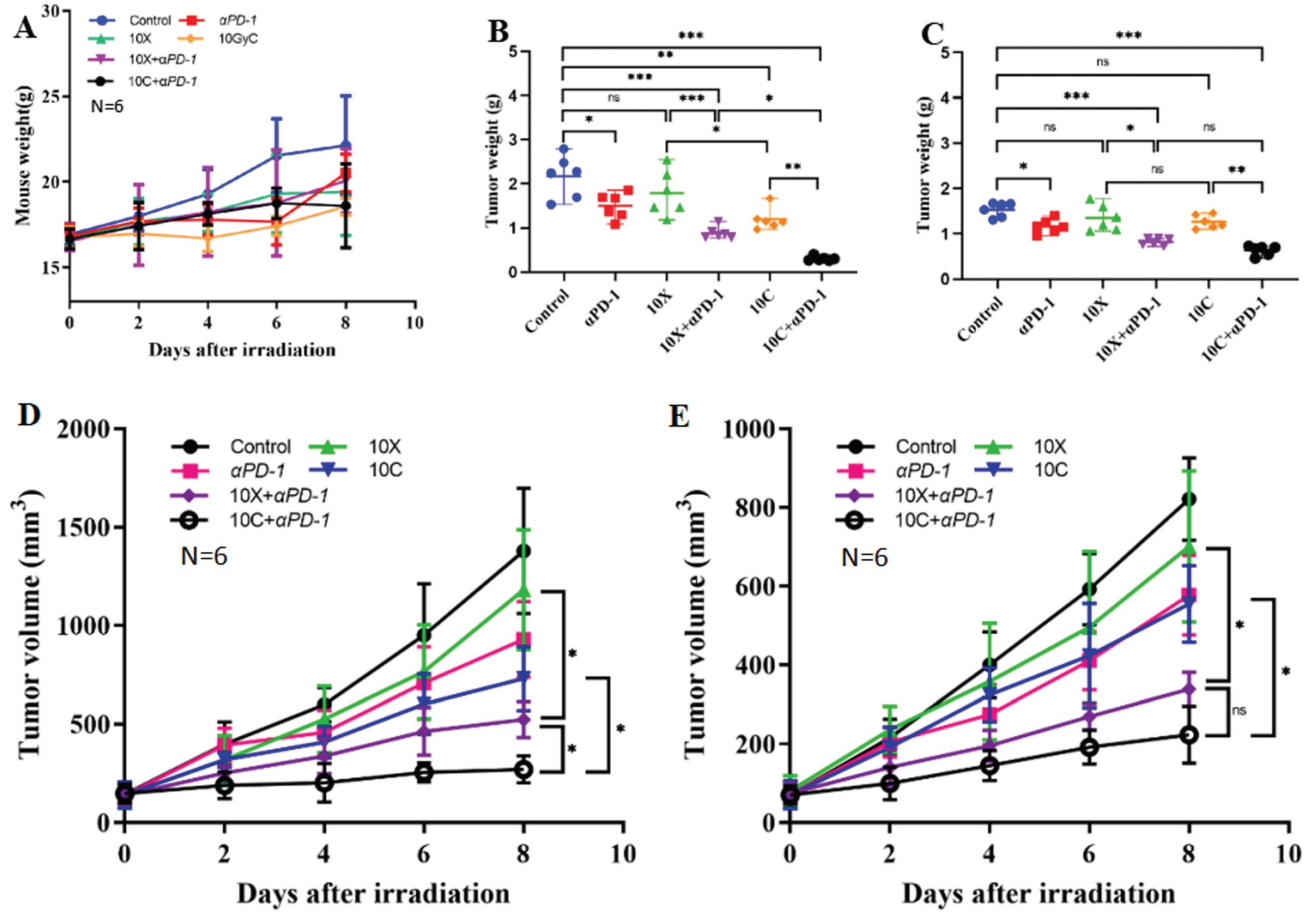
Changes in body weight, tumor weight and volume of mice in different intervention groups (A. Changes in average body weight of 6 mice. B. Changes of average tumor weight in irradiated area. C. Changes of average tumor weight in non-irradiated area. D. Changes of average tumor volume in irradiated area. E. Changes of average tumor volume in non-irradiated area. **p < 0.01, ***p < 0.001, ns *p* > 0.05).

Throughout the monitoring period, there were no fatalities in any of the therapy groups. Different groups of tumor-bearing mice had various changes in their weight. Using D0 as the radiation treatment day, the weight of the tumor-bearing mice in the control group continuously grew until it reached 22.13±2.92g at D8. The weight gain of the 10C group and 10C+PD-1 group was slower than that of the control group. At D8, the weights in the 10C group were 18.57±0.49g and 18.6±2.45g, respectively, although there was no discernible difference between the two groups when compared to the Control group (*p*>0.05). At D8, the weights were 19.42±2.56g in the 10X group, 20.07±1.90g in the 10X+PD-1 group, and 20.52±1.10g in the PD-1 group. Weight across the groups did not vary statistically significantly (*p*>0.05) (Fig. 4 A).

### Changes of tumor weight

At day eight, the mice in each group were killed, and the bilateral tumor tissues were taken out. After weighing, it was discovered that the weight of the tumors varied somewhat across the groups. In the control, PD-1, 10X, 10C, 10X+PD-1, and 10C+PD-1 groups, the tumor weight on the irradiated side was 2.168±0.476g, 1.495±0.290g, 1.791±0.517g, 1.198±0.245g, 0.886±0.136g, and 0.303±0.051g, respectively. In the control, αPD-1, 10X, 10C, 10X+αPD-1, and 10C+αPD-1 groups, the tumor weight of the non-irradiated side was 1.532±0.157g, 1.166±0.151g, 1.350±0.288g, 1.265±0.145g, 0.825±0.072g, and 0.635±0.108g, respectively. Both the 10C+αPD-1 group and the 10X+αPD-1 group substantially reduced (*p*<0.05) the tumor weight of the irradiated side and non-irradiated side as compared to the control group. When compared to the control group, the tumor weight on both sides in the PD-1 group was considerably reduced (*p*<0.05). In the 10C group, the tumor weight on the radiation-treated side reduced considerably (*p*<0.05), whereas the non-irradiated side did not alter significantly (*p*>0.05). The 10X group's bilateral tumor weight showed no discernible change (*p*>0.05). The tumor weight of both irradiated and non-irradiated side tumors in the 10X+αPD-1 group was considerably lower than in the αPD-1 group and 10X group (*p*<0.05). The weight of the bilateral tumors in the 10C+αPD-1 group was considerably reduced (*p*<0.05) when compared to the αPD-1 group and 10C group. On the irradiated side, the tumor weight in the 10C+αPD-1 group was considerably lower (*p<*0.05) compared to the 10X+αPD-1 group, while on the non-irradiated side, the two groups were comparable (*p*>0.05) (Figure 4 B-C). The combination of αPD-1 with either X-ray or carbon ion significantly inhibited the growth of bilateral tumors, with carbon ion significantly outperforming the X-ray combined group (*p*<0.05).

### Changes of tumor volume

The bilateral tumor volumes of mice in each group changed with time. The tumor volumes at D8 on the irradiated side were 1378.6±317.45 mm^3^, 929.53±193.38 mm^3^, 1180.91±304.17mm^3^, 731.85±163.02mm^3^, 524.00±90.54mm^3^, and 270.68±68.37 mm^3^ in control, αPD-1, 10X, 10C, 10X+αPD-1 and 10C+αPD-1 group respectively. The tumor volumes at D8 on the non-irradiated side were 820.99±104.97mm^3^, 577.17±100.66mm^3^, 700.36±191.97mm^3^, 554.29±97.06mm^3^, 349.84±48.15mm^3^, 223.2±71.96mm^3^ respectively in control, αPD-1, 10X, 10C, 10X+αPD-1and 10C+αPD-1 group. Compared with the Control group, the tumors volume on the irradiated side and non-irradiated side were significantly decreased in 10C+αPD-1 and 10X+αPD-1 group (*p*<0.05). Compared with the 10X+αPD-1 group, the tumor volume of 10C+αPD-1 group on the irradiated side was smaller, and the difference was statistically significant (*p*<0.05), but there was no significant difference between the two groups on the non-irradiated side (*p*>0.05). Compared with αPD-1 group and 10X group, the volume on bilateral tumors in 10X+αPD-1 group was significantly reduced (*p*<0.05). Compared with αPD-1 and 10C group, the tumor volume on both sides in 10C+αPD-1 group was significantly reduced (*p*<0.05). Compared with 10X group, there was no significant difference in tumor volume between the both sides of 10C group (*p*>0.05) (Fig. 4 D-E).

### Expression of TREX1, PD-L1, IFN-β and CD8+T cell infiltration in mouse tumor tissues

**Figure 5:**
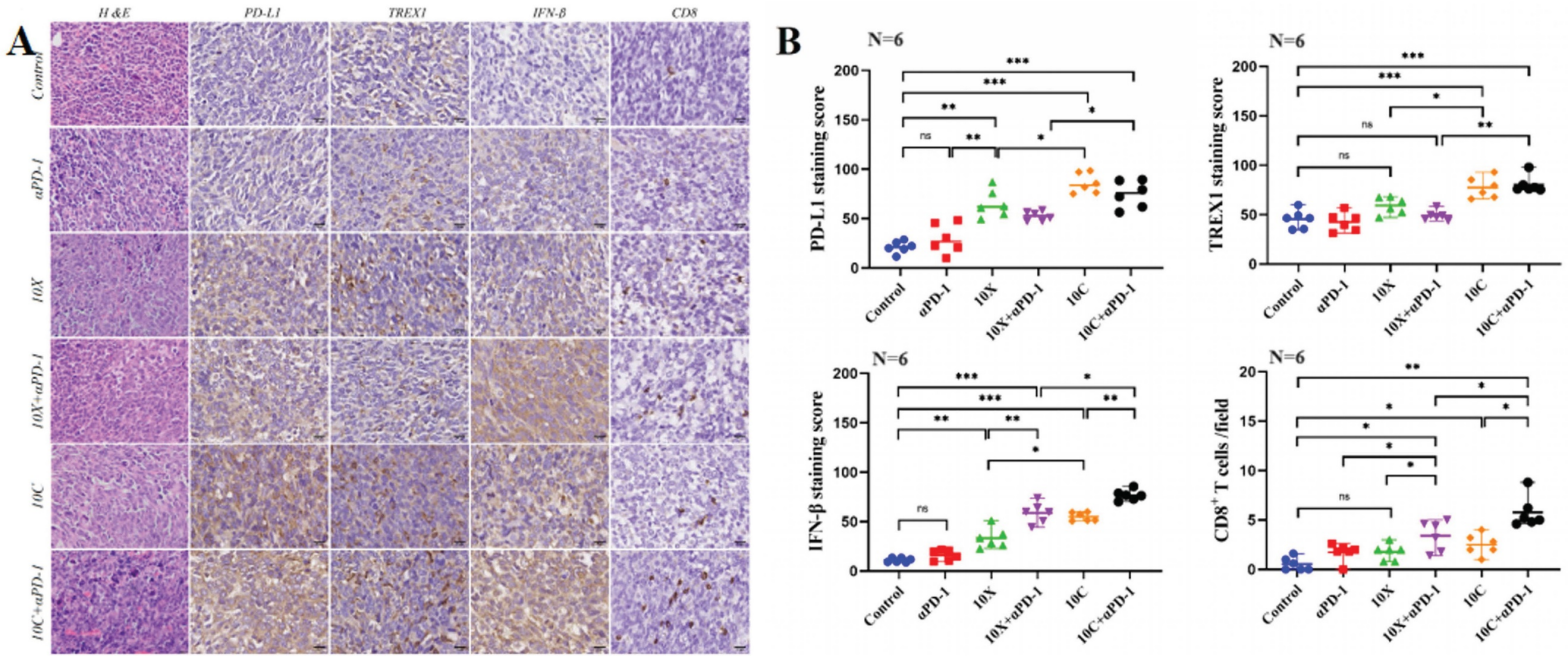
Effects of carbon ion or X-ray combined with PD-1 inhibitors on the expression of immune-related molecules in irradiated tumor tissues. A is the representative figure of hematoxylin and immunohistochemical staining (PD-L1, TREX1, IFN-β, CD8+T) on the irradiated side tumor (Multiple 400X). B were tumor immunohistochemical staining score (PD-L1, TREX1, IFN-β) and CD8+T cell count (*p < 0.05, **p < 0.01, ***p < 0.001, ns *p* > 0.05).

**Figure 6:**
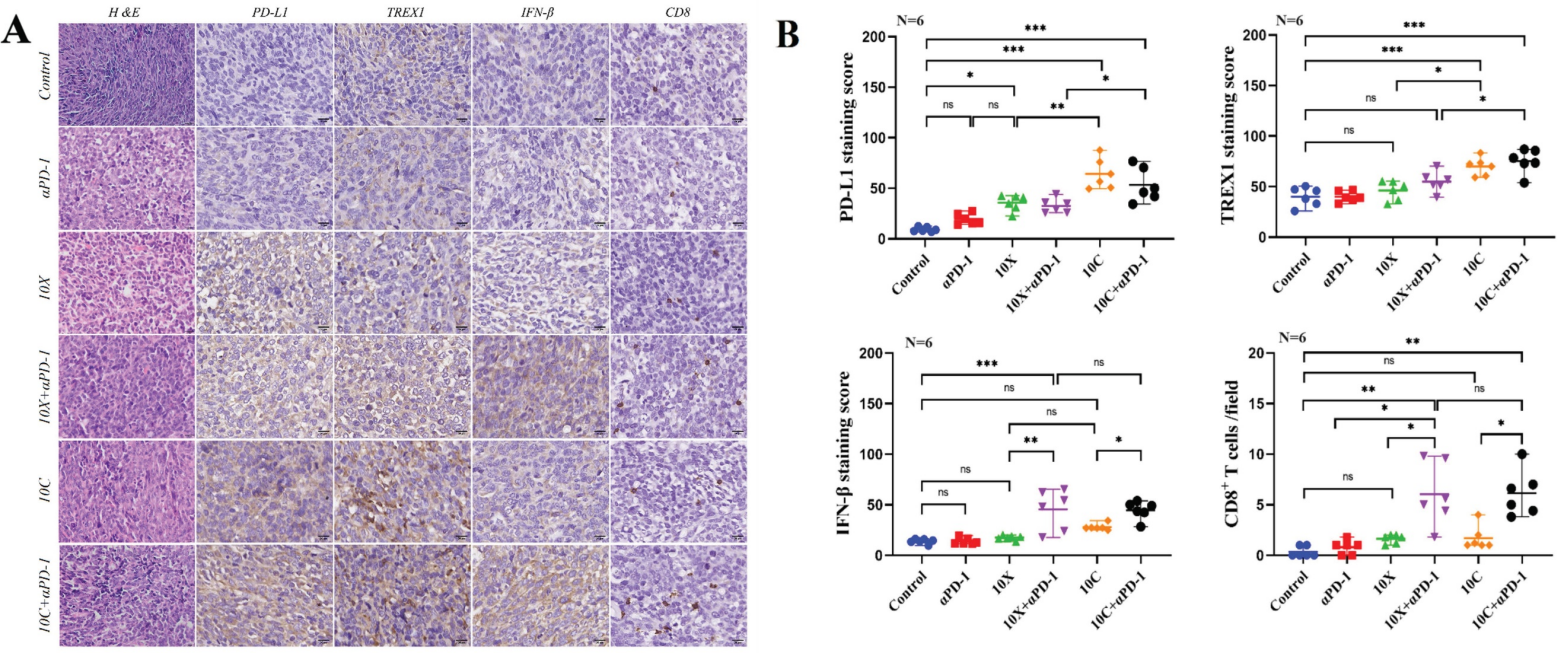
Effects of carbon ion or X-ray combined with PD-1 inhibitors on the expression of immune-related molecules in non-irradiated tumor tissues. A is the representative figure of hematoxylin and immunohistochemical staining (PD-L1, TREX1, IFN-β, CD8+T) on the non-irradiated side tumor (Multiple 400X). B were tumor immunohistochemical staining score (PD-L1, TREX1, IFN-β) and CD8+T cell count (*p < 0.05, **p < 0.01, ***p < 0.001, ns *p* > 0.05).

After the mice were killed, the expression of TREX1, IFN-β, PD-L1, and CD8+T cell infiltration in tumor tissue was found in order to investigate the effects of carbon ion or X-ray coupled PD-1 inhibitor on the immune milieu of cancers in vivo. Fig. 5-6 showed the IHC results and staining score of the irradiated and non-irradiated tumors. Compared with the control group, there were no significant differences in the expression of IFN-β, PD-L1, TREX1 and the infiltration ratio of CD8+T cells both in in αPD-1 group (*p*>0.05), expression of αPD-L1 in the 10X group was significantly increased both in both sides (*p*<0.05), expression of IFN-β was only significantly increased in irradiated side (*p*<0.05), and the expression of TREX1 and the infiltration ratio of CD8+T cells was not significantly increased in both sides (*p*>0.05). Compared with the control group, the expression of IFN-β and the infiltration ratio of CD8+T cells in irradiated side in 10C group were significantly increased (*p*<0.05), but there was no significant change in tumors of non-irradiated side (*p*>0.05). The expression of PD-L1 and TREX1 in bilateral tumors in 10C group were significantly increased (*p*<0.05). The expression of IFN-β and infiltration ratio of CD8+T cells in bilateral tumors were significantly increased in 10X+αPD-1 and 10C+αPD-1 groups. The expression of IFN-β and infiltration ratio of CD8+T cells in bilateral tumors were significantly increased in 10X+αPD-1 and 10C+αPD-1 groups. In the irradiated tumors, the infiltration ratio of CD8+T cells and the expression of IFN-β were higher in the 10C+αPD-1 group than in the 10X+αPD-1 group. The infiltration ratio of CD8+T cells and the expression of IFN-β were not significantly different between the two groups in the non-irradated tumors (*p*>0.05). In general, X-ray or carbon ion combined with αPD-1 significantly enhanced the immune response in the irradiated and non-irradiated tumors, and carbon ion combined with αPD-1 was more dominant in the irradiated field.

### Changes of CD3+T and CD3+CD8+T lymphocyte subsets in peripheral blood of mice between groups

**Figure 7:**
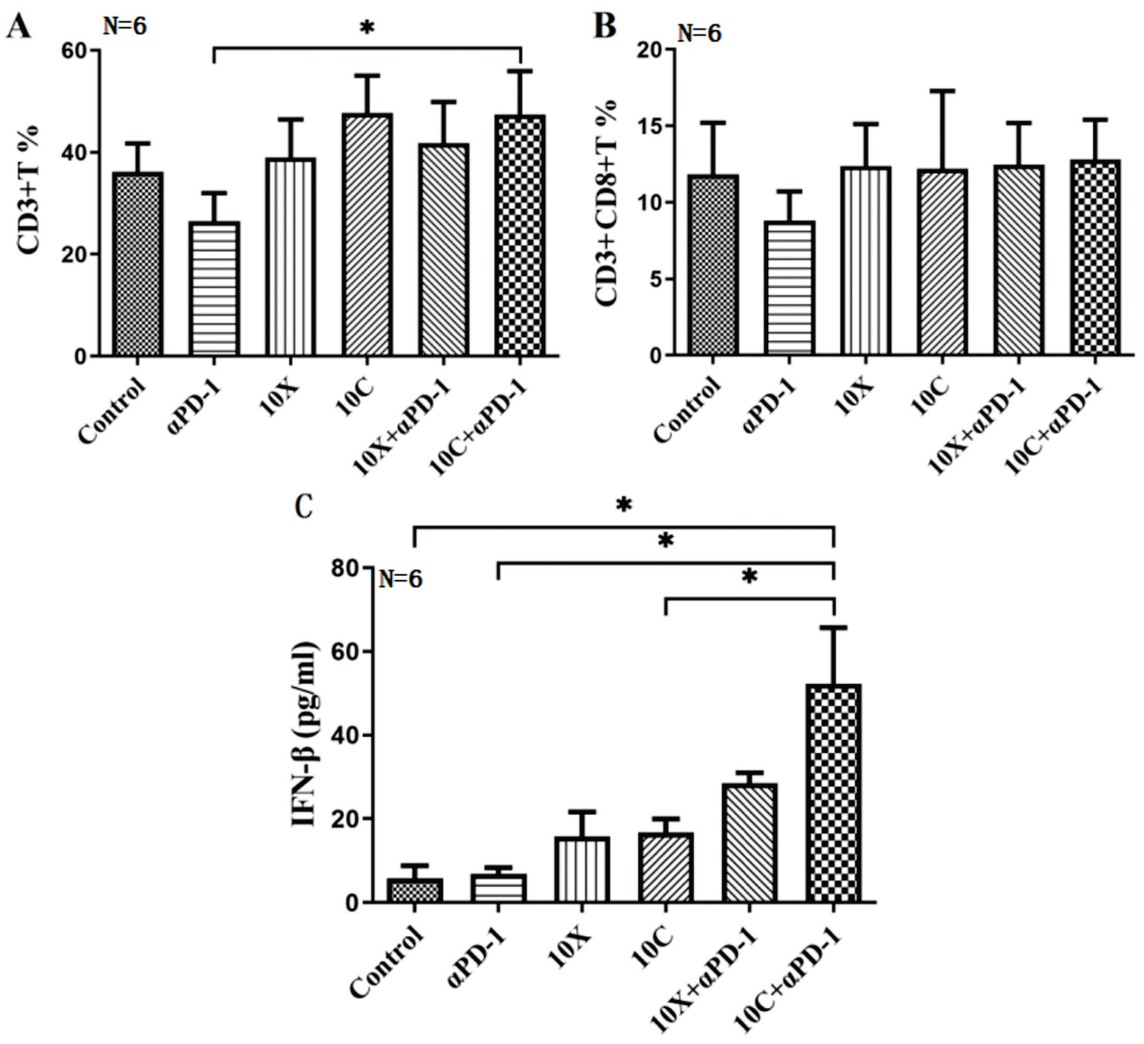
Changes of T lymphocyte subsets and IFN-β in peripheral blood of mice in each group (A. CD3+T lymphocyte, B. D3+CD8+T lymphocyte, C. IFN-β, * p < 0.05).

At D8, C57BL/6 tumor-bearing mice were examined using flow cytometry to identify total T lymphocytes and cytotoxic T lymphocyte subsets (CD3+T and CD3+CD8+T lymphocyte cell subsets). The findings indicated that following irradiation, the percentage of CD3+T lymphocyte cells rose, although to a different extent. There was no discernible change in the percentage of CD3+T cell lymphocytes in any group as compared to the Control group. The percentage of CD3+T lymphocyte cells in the 10C+αPD-1 and 10C groups was considerably higher than in the αPD-1 group (*p*<0.05). CD3+CD8+T lymphocyte cells seem to decrease in the αPD-1 group compared to the control group, but there is no discernible difference in the X-ray and carbon ion coupled with αPD-1 inhibitor group (Fig. 7A-B).

#### Changes of IFN-β in serum of mice

IFN-β concentrations in mouse serum varied significantly across groups (*p*<0.05). The serum IFN-β level in the 10X and 10C groups rose after irradiation compared to the control group, although this rise was not statistically significant (*p*>0.05); The serum IFN-β level in X-ray or carbon ion combined with αPD-1 group was significantly increased (*p*<0.05), especially in 10C+αPD-1 group (*p*<0.05); IFN-β levels were similar between αPD-1 group and control group (*p*> 0.05) (Fig. 7C).

### Effects of different doses of X-ray and carbon ion on PD-L1, IFN-β and TREX1 in Lewis cells

**Figure 8:**
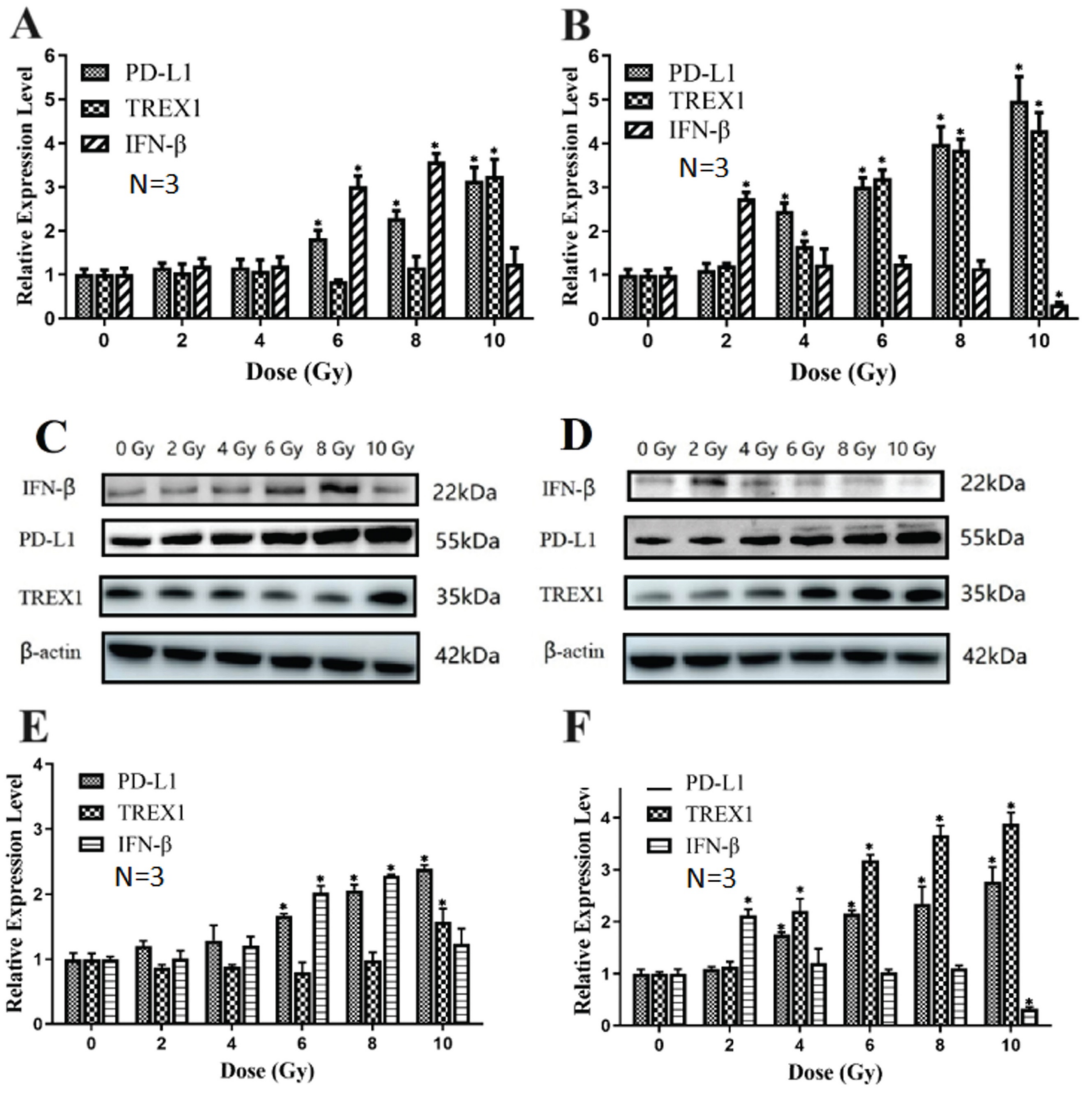
Effects of X-ray and carbon ion irradiation on mRNA and protein levels of PD-L1, IFN-βand TREX1 molecules (A. Effects of X-ray on PD-L1, IFN-βand TREX1 mRNA level in Lewis cells; B. Effects of carbon ion on PD-L1, IFN-β and TREX1 mRNA level in Lewis cells; C&E. Effects of X-ray on the expression of PD-L1, IFN-β and TREX1 proteins in Lewis cells; D&F. The effects of carbon ion rays on the expression of PD-L1, IFN-β and TREX1 proteins in Lewis cells; * *p* < 0.05, **p < 0.01).

In a dose-dependent way, X-ray irradiation may raise PD-L1 transcriptional level expression compared to 0Gy group. When compared to the 0Gy group, X-ray irradiation increased the expression of PD-L1 mRNA, and there were significant differences among 6Gy, 8Gy and 10Gy groups (*p*<0.05). X-ray irradiation of 6Gy and 8Gy significantly increased the expression of IFN-β mRNA (*p*<0.05). Only 10Gy X-ray significantly up-regulated TREX1 mRNA levels (*p*< 0.05) (Fig. 8A). Carbon ion irradiation also increased the expression of PD-L1 at the transcriptional level in a dose-dependent manner, and the expression of PD-L1 at 4, 6, 8, and 10Gy doses was significantly higher than that at 0Gy dose (*p*<0.05). Compared with 0Gy group, TREX1 mRNA expression increased significantly after 4, 6, 8 and 10Gy carbon ion irradiation. However, only the expression of IFN-β increased significantly after 2Gy carbon ion irradiation (*p*<0.05). The expression of IFN-β mRNA in Lewis cells irradiated with 10Gy carbon ion decreased significantly (*p*<0.05) (Fig. 8B).

Western blotting was used to find the variations in protein concentrations. Compared with the 0Gy control group, TREX1 expression was significantly up-regulated by carbon ions at 4, 6, 8, and 10Gy (*p*<0.05), while TREX1 expression was significantly increased by X-ray only at 10Gy (*p*<0.05). At the same physical dose, the ability of 2Gy carbon ion beam to induce Lewis cells to produce IFN-β was significantly stronger than that of 2Gy X-ray (*p*<0.05). The expression trend of PD-L1 at the translational level was similar to that at the transcriptional level for both X-ray and carbon ion (Fig. 8 C-F).

### Effects of irradiation on proliferation and apoptosis of Lewis cells after TREX1 gene silencing

**Figure 9:**
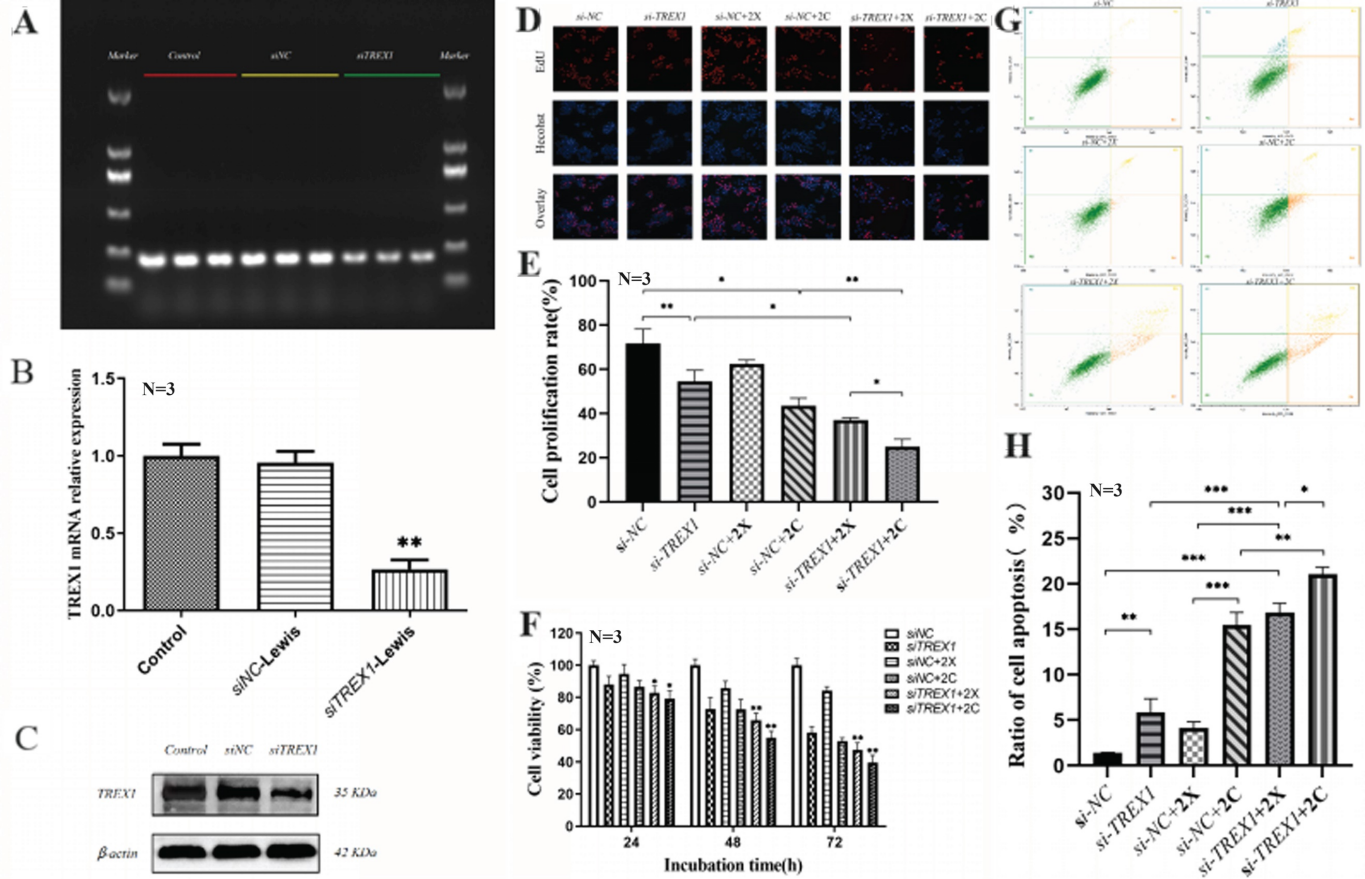
Effects of irradiation on proliferation and apoptosis of Lewis cells after TREX1 gene silencing (A. Agarose gel electrophoresis after TREX1 gene silencing; B. The transfection efficiency of siTREX1-Lewis was verified by Western blot; C. Protein immunoblotting of TREX1; D-F. The changes of cell proliferation and viability over time in each group, D was Edu representative image, E was Edu cell proliferation rate, F was CCK8 cell viability; G-H. The changes of cell apoptosis rate in each group; *p < 0.05, **p < 0.01, ***p < 0.001).

When Lewis cells' TREX1 gene was silenced by siRNA, TREX1 expression was around 30% lower than in the negative control group, meaning that the interference rate increased to 70%. Western blotting revealed the same pattern, showing that siTREX1-Lewis cells were successfully generated (Fig. 9 A-C).

Lewis cells' capacity to proliferate was shown to be considerably hindered by a dosage of >2Gy carbon ion radiation in the preliminary experiment, making it impossible to see the impact of the TREX1 gene deletion. To determine the proliferation activity of the two types of Lewis cells after 24hours, 48 hours, and 72 hours following irradiation, respectively, 2Gy X-ray and 2Gy carbon ion irradiation were used. The findings demonstrated that Lewis cell proliferation was significantly inhibited by the silencing of TREX1. Irradiation after silencing TREX1 could considerably decrease the proliferation of Lewis cells, and this impact was correlated with the kind of rays and the amount of time after irradiation. 2Gy X-ray and 2Gy carbon ion could both inhibit the proliferation of Lewis cells. After 2Gy X-ray irradiation, Lewis cell vitality was not considerably suppressed, and in fact, after 72 hours of irradiation, cell vitality tended to increase. However, following 2Gy carbon ion irradiation, cell vitality continued to decline, demonstrating a greater inhibitory impact. The findings imply that carbon ion is more effective than other irradiations in dramatically inhibiting Lewis cell growth when TREX1 is silenced (Fig. 9 D-F).

After being exposed to 2Gy X-ray and 2Gy carbon ion radiation, Lewis cells and siTREX1-Lewis cells were examined using flow cytometry to determine changes in apoptosis. We discovered that 2Gy carbon ion may better than X-ray induce Lewis cell apoptosis. Lewis cells were more likely to undergo apoptosis when TREX1 was silenced compared to the control group. When Lewis cells were exposed to iradiation after TREX1 was silenced, the rate of apoptosis was also increased dramatically (Fig. 9 G-H).

### Changes of IFN-β in si-RNA TREX1 Lewis cells with carbon ion irradiation

IFN-β levels changed after carbon ion radiation of 10 Gy was applied to siTREX1-Lewis and Lewis cells. It was discovered that siTREX1-Lewis cells had enhanced IFN-β expression, and that the rise had become more pronounced after 10Gy carbon ion radiation.

## Discussion

In order to simulate the clinical impact of local lesion irradiation coupled with PD-1 inhibitor on non-irradiated distant metastatic locations and to investigate associated mechanisms though alterations in TREX1, IFN-β, and PD-L1 in TME, a bilateral tumor bearing model of Lewis lung cancer was created. The distant tumor regression brought on by radiation treatment out in the field was referred to as the "abscopal effect" by Mole et al. in 1953, but this phenomenon became more pronounced after the advent of immunotherapy 16. The primary way that radiation-induced abscopal effect works is by directly killing local tumors, which releases tumor antigen, creates an in-situ tumor vaccine, activates the immune system, changes tumor micro-environment (TME) from immune desert type to immune hot type 17, and ultimately triggers an immune response to unirradiated tumors. However, only a small number of case reports in clinical trials have shown abscopal effects brought on by radiation alone. Combining radiation with immunotherapy, as opposed to radiotherapy alone, provides the potential to enhance the incidence of adverse effects in the age of immunotherapy. It is unknown if immunotherapy combined with carbon ion beam, which are high LET ray, would result in increased abscopal effects.

According to the study's findings, PD-1 inhibition in combination with either carbon ion beam or X-ray had a substantial inhibitory effect on tumors that had been exposed to radiation as well as those that had not, with carbon ion having a stronger impact on radio-exposed tumors. In both irradiated and non-irradiated tumors, immunohistochemical results showed that X-ray or carbon ion combined with PD-1 inhibitor could effectively induce the up-regulation of IFN-β and the increase of CD8+T cell infiltration in the tumor. The effect of carbon ion is more pronounced in irradiated tumors. However, as observed in the 10Gy carbon ion irradiation group, the expression of PD-L1 and TREX1 in both malignancies considerably increased following heavy dose carbon ion irradiation.

Our cell experiment results likewise supported the aforementioned conclusion. Under the same physical dosage, it was investigated how various LET rays affected the upstream regulatory factors TREX1, IFNB1, important marker of immunogenic death, and PD-L1 in Lewis lung adenocarcinoma cells. From the mRNA and protein levels, CD274(PD-L1), IFNB1, and TREX1 alterations were identified. According to the findings, CD274, IFNB1, and TREX1 all underwent changes with various radiation dosages, particularly CD274. Both X-ray and carbon ion were able to increase CD274 expression in cells in a dose-dependent way, and the carbon ion group experienced a more pronounced alteration. Only at a dosage of 10Gy did an X-ray considerably increase the expression of TREX1 compared to the 0Gy group. The expression of IFNB1 was negatively correlated with TREX1 expression. Furthermore, by deleting the TREX1 gene and exposing the cells to 10Gy of carbon ion radiation, the expression of IFNB1 was restored. It is hypothesized that a certain dosage threshold is necessary to induce TREX1 expression, and that various LET rays have different thresholds. Additionally, it is believed that TREX1 regulates IFNB1 expression. It was discovered that down-regulating TREX1 might reduce tumor growth, trigger tumor cell death, and improve the sensitivity of certain radiation. In light of the aforementioned findings, further research on radiation-induced anti-tumor immunity is still required. TREX1 may be one of the targets for upcoming radiotherapy and immunotherapy combinations since the optimum dose-fraction model has varied thresholds under various radiation doses.

Numerous studies have supported TREX1's role as a negative immunological regulator, reducing immunogenicity by destroying DNA that accumulates in the cytosol after radiation^18^. Furthermore, it has been discovered that single radiation doses with a threshold range from 12 to 18Gy promote its expression at levels adequate to destroy cytosolic DNA in several mouse and human cancer cells. The accumulation of IFN-stimulatory DNA in the cytoplasm of radioactively irradiated cells and the subsequent formation of anti-tumor T cell responses. Carbon ion, as a type of high LET ray, can cause more complex DNA damage. In this study, TREX1 showed significant increase under the irradiation is determined by the equilibrium between levels of dsDNA and TREX1. These data suggest a link between the immune-stimulatory effects of radiation and the DNA damage response, which is mediated via canonical pathways that regulate autoimmunity and the response to viral infections 19,20. High LET ray in the form of carbon ion may damage DNA in a more complicated way. In this work, TREX1 significantly increased after receiving a 10Gy dose of carbon ion. Tumor shrinkage on the irradiated and non-irradiated sides was not statistically significant when paired with PD-1 inhibitor, further demonstrating that there was a dosage threshold for the occurrence of the abscopal effect. On the other hand, the radiation-induced anti-tumor immunological response is considerably reduced after this dosage threshold is reached. This explains why the incidence of the genuine abscopal effect in clinical practice is so low.

ICD is a crucial mechanism to start the anti-tumor immune response and is triggered by several anti-cancer means. However, the ICD indicators shown by various treatment modalities vary. The major indicators of radiation-induced ICD include ATP, CRT, HMGB1, interleukin-1, and IFN-β 21. Among them, Type I IFN plays a key role, and when irradiation is used in conjunction with immunotherapy, Type I IFN is particularly important. In a study involving a tumor-bearing animal model with PD-1 inhibitor resistance, radiotherapy and PD-1 inhibitor were administered simultaneously to block the IFN-β pathway, and it was discovered that this combination was unable to exhibit the original anti-tumor^22^. This research reveals that radiation promotes anti-tumor immune response reversal resistance in PD-1 inhibitor-resistant tumors by stimulating the type I IFN signaling pathway to increase IFN-β expression rather than IFN-γ. Formenti et al.^23^ discovered that the use of CTLA-4 inhibitors in combination with radiotherapy could enhance the anti-tumor immune response in vivo. Additionally, they discovered that the increase in serum IFN-β and the number of blood T cells following radiotherapy were highly correlated with the occurrence of the immune response, suggesting that IFN-β could predict the efficacy of the anti-tumor immune response. IFN-β may be employed as one of the ICD indicators of an anti-tumor immune response based on the aforementioned findings.

According to the study's findings, both irradiated and non-irradiated tumors in the combined treatment group had significantly higher levels of the ICD marker IFN-β than the non-combined group did. It was also clear that carbon ion was superior to X-ray at triggering the anti-tumor immune response. IFN-β was considerably upregulated in both the irradiated tumor and abscopal tumors in the 10C+ɑPD-1 group, with its serum concentration being the greatest. This shows that combination therapy, particularly the involvement of high-LET rays, not only effectively enhances the immune effect of the irradiated tumor but also stimulates the immune response products in the circulatory system, which is a process from the local to the circulatory system and ultimately to all parts of the body, and realizes the immune response to the abscopal tumor. carbon ion may be a superior option for combination immunotherapy due to possessing characteristics that make them more effective than conventional radiation in triggering the body's anti-tumor immune response.

Many studies on tumor bearing animal models also showed similar results. Ando et al. 10 compared the efficacy of carbon ion or X-ray radiotherapy combined with DC immunotherapy using a mouse lung metastasis model of squamous cell carcinoma with NR-S1, and the results suggested that compared with X-ray radiotherapy alone, immunotherapy alone and carbon ion radiotherapy alone, carbon ion combined with DC immunotherapy could significantly reduce the number of lung metastases. At low doses (2-4 Gy), carbon ion radiation and DC immunotherapy may dramatically reduce lung metastasis, but high doses (15 Gy) of X-ray and DC immunotherapy can greatly reduce lung metastasis. At the same time, carbon ion exposure dramatically increased the expression level of CRT in NR-S1 cells, indicating that the carbon ion had a stronger activation of the anti-tumor immunological response at the same BED. By enhancing the production of CRT and stimulating the maturation of DC to trigger an anti-tumor immune response, carbon ion irradiation in combination with intravenous injection of DC may improve the ICD of tumor cells and suppress unirradiated abscopal metastases. Takahashi et al.^24^ utilized an LM8 mouse osteosarcoma with double hindlimb tumor bearing model to investigate radiation-immune effects. All test animals were divided into four groups, control, carbon ion-irradiated unilateral hindlimb tumor, PD-L1+CTLA-4 inhibitor alone (PD-L1+CTLA 4 group), and carbon ion-irradiated unilateral hindlimb tumor coupled with PD-L1+CTLA 4 group (Comb group). The findings demonstrated that CD8+T and other immune cells in the Comb group were much more activated than those in the other groups. Additionally HMGB-1 expression and release were likewise significantly elevated in the Comb group. In comparison to 20% in the PD-L1+CTLA 4 group, 64% of mouse in the Comb group exhibited a complete response in their non-irradiated tumor, and their overall survival was also much longer. Both irradiated primary tumors and non-irradiated distant metastases exhibited significantly higher antitumor immune effectiveness when carbon ion and ICI were used in combination. In a study by Matsunaga et al.^25^, the efficacy of carbon ion combined with DC was compared in squamous cell carcinoma bearing model with low immunogenicity SCCVII with nude mouse. The results suggested that carbon ion combined with DC could significantly reduce both primary and metastatic tumors in the mouse model with low immunogenicity.

Our findings also imply that a greater single fraction dosage may not be theoptimal choice for the combination of radiation and immunotherapy. In this investigation, following exposure to 10Gy X-ray or 6, 8, or 10Gy carbon ion, the expression of TREX1 was dramatically up-regulated whereas the expression of IFN-β, an immunogenic marker, was lowered. In vivo investigations likewise had similar outcomes. In the melanoma mouse model, a single irradiation of 7.5Gy or 10Gy could activate the anti-tumor immune response of the body, but the increase of Treg in the spleen might inhibit the tumor-specific immune response when a large dose of 15Gy or above was applied^26^, ^27^. There is apparently a relationship between the dose fraction pattern and the anti-tumor immune response brought on by high LET rays. We found there was high expression of TREX1 at a particular dosage range in Lewis cells in this research, IFN-β expression rose as the irradiation dosage increased within a certain range, but when TREX1 expression started to grow at a greater dose, IFN-β expression stopped increasing and even drastically dropped. In both the X-ray and carbon ion groups, the expression level of PD-L1 progressively rose with an increase in dosage, but the threshold values for a significantly higher level of PD-L1 expression in each group were different. According to these findings, distinct LET rays might diminish or strengthen their immunogenicity at certain dosages, although the threshold for regulating immunogenicity varied between two types of rays.

Another important indicator for demonstrating the presence of cell ICD is CRT translocation to the surface of the cell membrane. Heavy ion may boost the production of ATP, HMGB1, CRT, and other DAMPs more than typical ray, which results in stronger tumor cell ICD, according to both cell and animal research. Huang et al. 28 showed that low dose (2Gy or 4Gy) carbon ion had stronger ICD-inducing ability than proton and photon of the same dose, but at high dose (10Gy), CRT expression level of tumor cells induced by carbon ion decreased compared with proton and photon of the same dose. The reason for the decrease of CRT level may be that the radiation dose exceeded the optimal dose window to induce ICD in tumor cells, which triggered a different cell death pattern than that of low dose. Zhou et al.^29^ demonstrated that a dose of 5Gy(RBE) of carbon ion irradiation induced ICD characterized by the exposure of calreticulin on the plasma membrane surface, the phosphorylation of eIF2a, the release of ATP into the extracellular space, the exodus of HMGB1 from the nucleus, and the induction of the Type I IFN response in vitro and in vivo. It is significant to note that, while having a lower dosage level, 5Gy (RBE) of CIRT was more successful in inducing tumor immunogenicity than 5Gy of X-ray. The effects of CIRT were further strengthened by the addition of anti-PD-1 treatment, which also elevated the expression of PD-L1 and greatly increased the expression of immune response markers in the blood, spleen, and tumors in vivo. This suggests that not only may different dose fractionation patterns cause various types and intensities of immune response, but also that the optimum threshold of the anti-tumor immune response produced by carbon ion radiation may vary from that of X-ray radiation. Hypofractionated radiation should be carefully evaluated in conjunction with immunotherapy, which is also consistent with our results in vivo of tumor-bearing animal model.

In conclusion, the combination of different LET ray and PD-1 inhibitor results in a greater anti-tumor impact and initiates an abscopal effect on Lewis lung adenocarcinoma-bearing mice via higher tumor immunogenicity and increased CD8+T infiltration in TME. Higher fractionated doses inhibited the anti-tumor immune effect by up-regulating TREX1 and decreasing IFN-β expression, which could serve as a prognostic marker and potential target of immunotherapy in patients with lung adenocarcinoma. However, carbon ion could activate anti-tumor immunity better than X-ray at the same appropriate single dose.

## Figures and Tables

**Figure 10:**
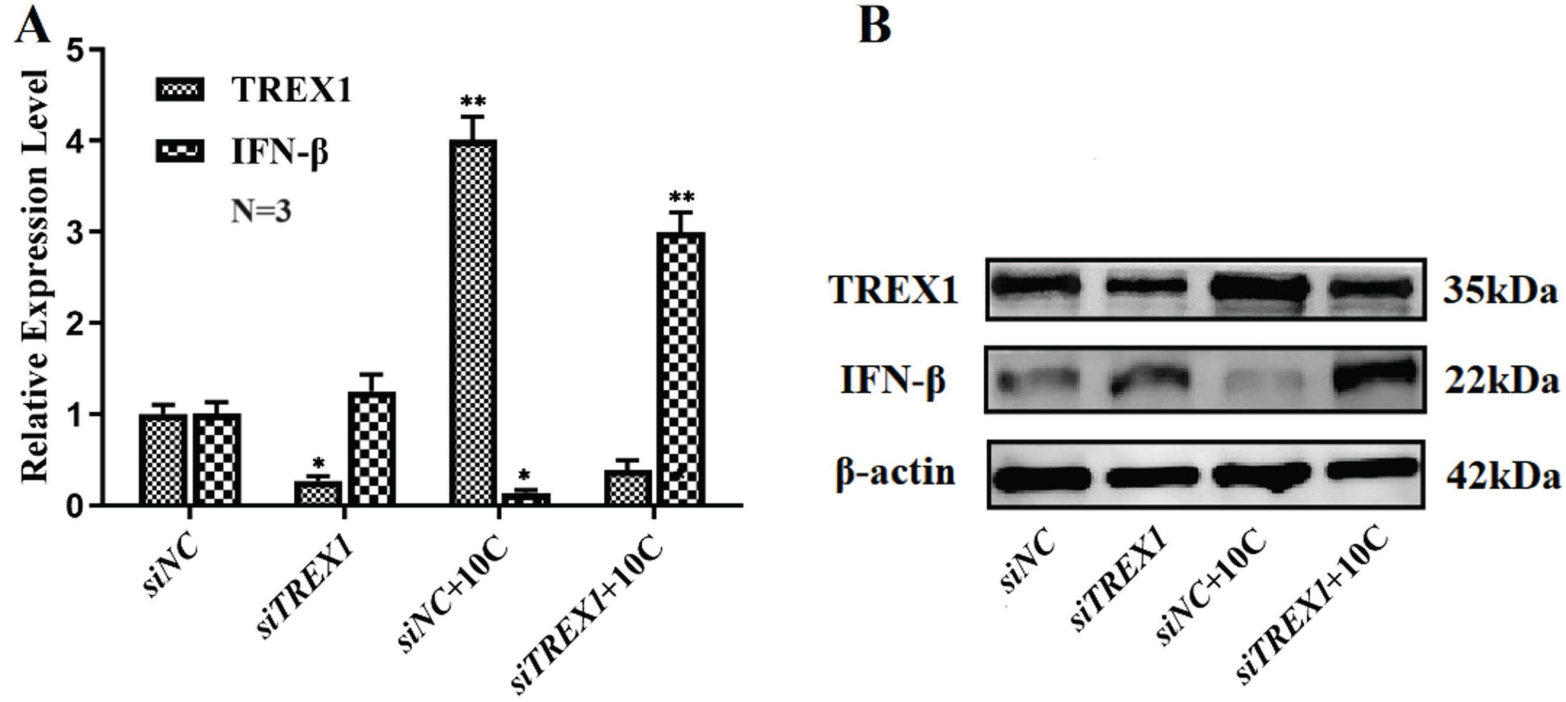
Changes of IFN-β and TREX1 in siTREX1 Lewis cells with 10 Gy carbon ion irradiation (A. The statistical results of qRT-PCR; B. Protein immunoblotting; *p<0.05).

**Table 1:** Primer sequence of target genes

Gene	Forward	Reverse	Length (bp)
*TREX1*	CAGACCCTCATCTTCTTAGACCT	CAGGGCTACAGGCTTTCCC	205
*PD-L1*	GCTCCAAAGGACTTGTACGT	TGATCTGAAGGGCAGCATTTC	238
*IFN-β*	CAGCTCCAAGAAAGGACGAAC	GGCAGTGTAACTCTTCTGCAT	138
*β-actin*	GAAGATCCTGACCGAGCGT	CCACAGGATTCCATACCCAA	249
